# Functional Coupling between DNA Replication and Sister Chromatid Cohesion Establishment

**DOI:** 10.3390/ijms22062810

**Published:** 2021-03-10

**Authors:** Ana Boavida, Diana Santos, Mohammad Mahtab, Francesca M. Pisani

**Affiliations:** 1 Istituto di Biochimica e Biologia Cellulare, Consiglio Nazionale delle Ricerche, Via P. Castellino 111, 80131 Naples, Italy; ana.boavida@ibbc.cnr.it (A.B.); diana.santos@ibbc.cnr.it (D.S.); md.mahtab@ibbc.cnr.it (M.M.); 2 Dipartimento di Scienze e Tecnologie Ambientali Biologiche e Farmaceutiche, Università degli Studi della Campania Luigi Vanvitelli, Via Vivaldi 43, 81100 Caserta, Italy

**Keywords:** DNA replication, replication proteins, sister chromatid cohesion, cohesin, cell cycle

## Abstract

Several lines of evidence suggest the existence in the eukaryotic cells of a tight, yet largely unexplored, connection between DNA replication and sister chromatid cohesion. Tethering of newly duplicated chromatids is mediated by cohesin, an evolutionarily conserved hetero-tetrameric protein complex that has a ring-like structure and is believed to encircle DNA. Cohesin is loaded onto chromatin in telophase/G1 and converted into a cohesive state during the subsequent S phase, a process known as cohesion establishment. Many studies have revealed that down-regulation of a number of DNA replication factors gives rise to chromosomal cohesion defects, suggesting that they play critical roles in cohesion establishment. Conversely, loss of cohesin subunits (and/or regulators) has been found to alter DNA replication fork dynamics. A critical step of the cohesion establishment process consists in cohesin acetylation, a modification accomplished by dedicated acetyltransferases that operate at the replication forks. Defects in cohesion establishment give rise to chromosome mis-segregation and aneuploidy, phenotypes frequently observed in pre-cancerous and cancerous cells. Herein, we will review our present knowledge of the molecular mechanisms underlying the functional link between DNA replication and cohesion establishment, a phenomenon that is unique to the eukaryotic organisms.

## 1. Introduction

Sister chromatid cohesion is the process by which duplicated DNA molecules are held together from the time of their synthesis in S phase till they separate in mitosis to ensure an even distribution of the genetic information to the daughter cells. A key player in this process is cohesin, a protein complex whose structure, DNA-binding mode, functions and regulation appear to be evolutionarily conserved throughout all eukaryotic organisms. Herein we summarize what is known about the functional link existing between DNA replication and sister chromatid cohesion establishment and how these important cellular processes are regulated. In this review we start with a general description of the cohesin architecture and the molecular mechanisms underlying cohesin loading onto DNA. Then, we illustrate the roles played by many DNA replication factors in assisting cohesion establishment. Lastly, we present and discuss models on how cohesin molecules are “re-cycled” at the replication forks and converted into a form able to stably tether newly duplicated DNA molecules.

## 2. The Cohesin Complex, Its Regulators and the Cohesin Cycle

In human somatic cells, the cohesin core complex consists of four subunits: Smc1 (for which two versions have been described Smc1A and Smc1B), Smc3, Scc1 and either SA1 or SA2 (also known as Stag1 and Stag2; Scc3 in yeast) depending on the cell types and/or developmental stages [1,2]. Smc proteins are long mostly helical polypeptides that fold back on themselves at a hinge domain forming a flexible and extended anti-parallel coiled coil structure. Their N- and C-terminal ends are juxtaposed and form together a globular nucleotide-binding domain (NBD) [3]. The coiled coil structure of the Smc1 and Smc3 proteins shows high conformational mobility, especially at a point, named “elbow”, that is located halfway along their length. The “elbow” flexibility confers cohesin ring the ability to fold back on itself, assuming different conformations during DNA loading/unloading process [4,5,6,7]. Smc1 and Smc3 form V-shaped dimers through a stable association of their hinge domains, whereas engagement of the NBDs is required for assembling a functional ATPase catalytic pocket, which has a sandwiched arrangement of nucleotide binding and hydrolysis sites, typical of the ATP-binding cassette (ABC) transporters. The Scc1 subunit (also named Rad21 or kleisin subunit) bridges the Smc1-Smc3 dimer forming a tripartite ring-like structure that is believed to topologically entrap sister chromatids in its interior. The fourth cohesin core complex subunit, SA1/2 (Stag1/2), is a dragon-shaped HEAT-repeat protein, which associates with the central portion of Scc1 through an extended interaction surface. SA1/2 binding to Scc1 strengthens the tripartite cohesin ring and serves as a binding platform for multiple cohesin regulators, as detailed below [1,2,3].

**Figure 1:**
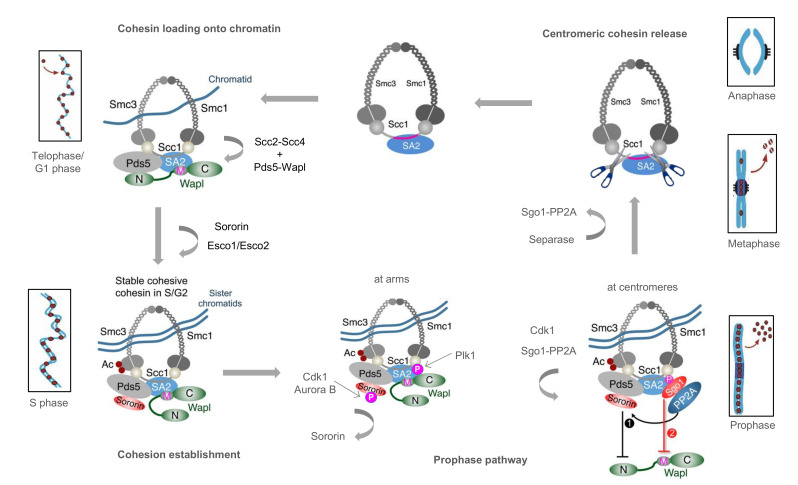
Cohesin cycle in human cells. The cohesin loader, Scc2-Scc4, and unloader, Pds5-Wapl, promote dynamic association of cohesin to chromatin in telophase and G1. During S phase, Smc3 acetylation and Sororin binding stabilize cohesin association to chromatin. Sister chromatids are entrapped by cohesin rings at the replication forks and cohesion is established. In prophase, cohesin bound to chromosome arms is released by Wapl-Pds5 following phosphorylation of Sororin (“prophase pathway”). In contrast, cohesin bound at the centromeres is protected by Shugoshin (Sgo1) and protein phosphatase 2A (PP2A) against the releasing activity of Wapl. At the metaphase-anaphase transition, Shugosin-PP2A dissociates from cohesin. Thus, in anaphase cohesin can be cleaved by Separase to enable chromosome segregation. Cohesin drawing has been inspired by [10] with modifications.

Cohesin functions are regulated by a set of accessory proteins, including the Scc2–Scc4 loader and the Pds5–Wapl releasing complex [3]. Scc2-Scc4 loads cohesin onto chromatin in telophase (in mammals) or in G1 phase (in yeast), prior to DNA replication. In G1 cohesin association with chromatin remains dynamic and chromatin-bound cohesin can be released by the Pds5-Wapl complex that promotes opening and release of cohesin rings [8]. Pds5 is stably associated with the cohesin Scc1 subunit, close to the Smc3-Scc1 interface. During S phase in mammalian cells Smc3 acetylation by Esco1 and Esco2 (Eco1 in yeast) enables binding to Pds5 of Sororin, an additional cohesin regulator that is exclusively present in metazoans. Sororin displaces Wapl from Pds5, but Wapl remains associated with cohesin through its interaction with SA1/2. This form of cohesin that contains Sororin, Pds5 and Wapl is believed to stably bind sister chromatids and establish cohesion in vertebrates during S phase [8]. When cells enter mitosis, phosphorylation of Sororin by mitotic kinases (CDK1 and Aurora B) disrupts the Sororin-Pds5 interaction allowing Wapl to re-engage Pds5 and trigger cohesin release from chromosome arms (“prophase pathway”). At the same time, cohesin bound at centromeres is protected from the releasing activity of Wapl by Shugoshin (Sgo1) and, thus, cohesin becomes resistant to the spindle-pulling force acting at sister kinetochores. This enables the generation of a kinetochore tension necessary for spindle-checkpoint inactivation. At the same time, Shugoshin migrates from centromeres to the kinetochores, leaving centromeric cohesin unprotected from the action of Separase, a protease that cleaves Scc1 at two specific sites. At the anaphase onset, centromeric cohesin is released and chromosome segregation can start. The alternance of cohesion establishment and dissolution defines the so-called “cohesin cycle”, a process that is strictly regulated by the cell cycle machinery jointly with a plethora of cohesin regulators (see Figure 1) [9].

The molecular mechanisms by which Scc2-Scc4 and Pds5-Wapl promotes association/dissociation of cohesin onto/from DNA have not yet been completely elucidated. Loading/unloading reactions were analyzed in vitro using budding yeast purified cohesin, Scc2-Scc4 and Pds5-Wapl complexes [11,12,13]. These elegant biochemical studies carried out by Murayama in Uhlmann laboratory revealed that both cohesin loading and releasing processes require the Smc1-Smc3 ATPase activity and suggest that chromatids may follow a similar path during transport into or out of the cohesin ring. In fact, in either directions DNA has to pass through two interlocking gates: one formed by the Smc NBDs (named “head gate”), which is opened/closed by an ATP-binding/-hydrolysis cycle, and the other one made by the Smc3-Scc1 interface (named “kleisin N-gate”), whose opening is regulated by the action of Pds5-Wapl. Besides, a critical role in these processes is also played by two Smc3 DNA-sensing Lysine residues (Lys105 and Lys106 in budding yeast Smc3) that trigger ATP hydrolysis in the presence of DNA. The Scc2-Scc4 loader complex is believed to stabilize cohesin in a folded conformation that exposes the Smc3 sensor Lysine residues enabling their interaction with DNA. During the sister chromatid cohesion establishment process these residues are acetylated by Eco1/Esco1-Esco2, a modification that inhibits the cohesin ATPase activity preventing ring opening. Thus, Smc3 acetylation counteracts the action of the Pds5-Wapl unloader ensuring stable association of cohesin with sister chromatids at the replication fork. Conversely, other studies revealed the importance of an additional cohesin gate, the one formed by the Smc hinge (named “hinge gate”) and suggested an alternative mechanism of DNA entrapment that involves opening of the “hinge gate” [14,15]. This model is mainly based on the observation that in budding yeast cells a rapamycin-mediated artificial fusion of Smc3 with Scc1, which blocks the “kleisin N-gate”, does not impair cohesin loading onto chromatin and sister chromatid cohesion; whereas mutations of conserved basic residues in the hinge region of yeast Smc1 and Smc3 subunits affect cohesin functions [14] in a way that seems to be conserved also in mammalian cells [16].

Structural analyses of the cohesin loader/unloader complexes have provided important clues on how cohesin ring opening/closing can be carried out. These studies revealed that Scc2 and Pds5 (and also SA1/2) are HEAT-repeat containing proteins adopting a similar elongated bent conformation with a terminal hook-like structure [8,17,18,19]. Scc2 and Pds5 are both able to interact with Scc1 and, interestingly, their binding surfaces on Scc1 overlap being located close to the Scc1-Smc3 interface. This led to the proposal that Pds5 and Scc2 can associate with cohesin in a mutually exclusive way. The function of Scc4, the other subunit of the cohesin loader complex, is less defined. It is believed that Scc4 could have regulatory functions and be responsible for recruiting the loader at different specific chromosomal locations, as described in more detail in the next Section.

Recent structural analyses based on cryo-electron microscopy have revealed the structure of the human and budding yeast cohesin-Scc2-DNA complex [6,7]. Both these studies have revealed that Scc2 (NIPBL in mammalian cells) interacts extensively with the cohesin complex and stabilizes it in a folded back conformation where different DNA-binding channels are formed/reshaped as a consequence of conformational changes induced by ATP-binding/hydrolysis. Of note, in the human ATP-bound cohesin-NIPBL-DNA complex structure the cohesin hinge region adopts an “open washer” conformation and docks onto the SA1 subunit, providing an important structural framework to the “hinge-gate” model [7]. These structural studies will help dissect mechanistic aspects of the cohesin DNA loading/unloading reaction. Besides, they will unveil the molecular bases of recently described non-canonical functions of the cohesin complex that are dependent on its ability to promote loop extrusion in an ATP-dependent manner [4,5,20,21].

## 3. Cohesin Recruitment at the Pre-Replication Complex (Pre-RC)

It has been shown that in the *Xenopus* egg extract system the Scc2-Scc4 cohesin loader is recruited to the pre-replication complexes (pre-RCs) through a direct interaction between Scc4 and Dbf4-dependent kinase (DDK; also known as Cdc7-Dbf4) [22,23,24]. Pre-RCs are formed in G1 through the sequential binding of the origin recognition complex (ORC), Cdc6, Cdt1 and the MCM2-7 complex to the origins of replication [25]. At the G1/S border pre-RCs are activated by DDK-mediated phosphorylation of MCM2-7, a modification that triggers binding of GINS and Cdc45 to form the Cdc45/MCM2-7/GINS, CMG, complex, the replicative DNA helicase, and establish active replication forks [26]. Since DDK associates with the pre-RCs during G1 phase, its interaction with the Scc2-Scc4 complex might be critical for loading cohesin at sites where replication forks will be established in the subsequent S phase. Interestingly, genome-wide chromatin immuno-precipitation experiments in *Drosophila* cell lines revealed a high concordance between binding sites of ORC and cohesin, suggesting that the pre-RC may be required for cohesin loading onto DNA [27]. Nevertheless, depletion of Cdt1, a component of the pre-RC, did not remarkably reduce association of cohesin to chromatin in *Drosophila* cells, arguing against that possibility. However, it is still plausible that inactivation of any of the pre-RC components can specifically reduce cohesin loading onto chromatin only in S phase, while a MCM2-7-independent cohesin loading mechanism would be active in telophase and G1. In fact, it has been proposed that cohesin loading might be also dependent on active gene transcription and require the DNA loop extrusion activity that has been recently described for the human Scc2-cohesin complex [4,5,28].

Nevertheless, in *Saccahromyces cerevisiae* cohesin loading onto DNA is independent from pre-RC formation, since cohesin association to chromatin is not affected in *CDC6* mutant cells unable to efficiently assemble the pre-RCs [29]. Besides, genome-wide chromatin binding analysis in budding yeast cells indicated that cohesin subunits and ORC binding sites do not coincide [30,31]. In *S. cerevisiae* Scc4 is critical for recruiting the Scc2-Scc4 loader complex to centromeres (*CEN* sites), where cohesin rings are actively loaded. Of note, it was demonstrated that Scc2-Scc4 directly interacts with the kinetochore protein Ctf19 at *CEN loci*. This association was found to be dependent on Ctf19 phosphorylation by DDK and to be important for centromeric cohesion [32]. Therefore, it seems that a DDK-dependent cohesin loading mechanism is conserved in all eukaryotic organisms. Nevertheless, an ortholog of the yeast Ctf19 kinetochore protein has not yet been identified in vertebrates, making it plausible that the cohesin loader complex of these organisms could recognize and bind DDK-phosphorylated sequence motifs of other not yet identified protein targets. Interestingly, a comprehensive analysis carried out in the Yu laboratory revealed that in synchronized HeLa cells cohesin is loaded onto chromatin by the Scc2-Scc4 loader complex at sites adjacent to those occupied by the pre-RCs in a process that is dependent on the activity of DDK [33].

## 4. Coupling between DNA Replication and Sister Chromatid Cohesion Establishment

Cohesion establishment requires the conversion of a “dynamic” form of cohesin bound to a single chromatid to a “cohesive” acetylated form of cohesin bound to the two sister chromatids. A natural context for this conversion is present at the replication forks, where the newly duplicated sister chromatids are transiently bridged by the DNA replication machinery. The existence of a functional coupling between chromosomal cohesion and replication is exemplified by the finding that, on one hand, down-regulating several cohesin regulators has consequences on replication fork dynamics [34,35,36] and, on the other hand, loss of many components of the replication machinery gives rise to sister chromatid cohesion defects [37,38]. A list of these replication proteins, named “cohesion establishment factors”, is reported in Table 1. It was proposed that some of these proteins (including the proliferating cell nuclear and antigen, PCNA, Ctf18, Mrc1/Claspin, Tof1/Timeless, Csm3/Tipin, Ctf4/AND-1 and the Chl1/DDX11 DNA helicase) play a role in recruiting regulating the acetyltransferases at the replication forks. Genetic analyses carried out in yeast revealed that none of these factors was essential for viability, but deletion of any of them caused a clear decrease in cohesin acetylation and defects in sister chromatid cohesion [39,40]. Besides, the above non-essential cohesion establishment factors have been grouped into two pathways based on their genetic interactions in *S. cerevisiae*: one group includes Ctf4, Tof1, Csm3 and Chl1 and the second RFC^Ctf18^, the alternative PCNA loader complex (see below for details), and Mrc1 [41]. The existence of two independent pathways responsible for cohesion establishment at the replication forks has been reaffirmed and more thoroughly investigated in a very recent study published by Nasmyth and collaborators [42]. These Authors have addressed the important question of whether pairing of the newly replicated chromatids is produced by conversion of cohesin rings preloaded onto the DNA template into a cohesive form (cohesin conversion/”re-cycling” mechanism) or by making use of the nucleoplasmic cohesin pool that has to be loaded *de novo* at sites of DNA synthesis (*de novo* loading mechanism). They have used an in-vivo cohesin conversion assay based on a yeast strain that harbored a temperature sensitive *SCC2* mutant allele (to block *de novo* cohesin loading at a non-permissive temperature) and expressed a cross-linkable form of cohesin containing a Separase-resistant Scc1 subunit. Taking advantage of the presence of a PK-tag on the Scc1 subunit, cross-linked cohesin rings could be immunoprecipitated from the cell extracts and their association with one (catenated monomers, CMs) or two mini-chromosomes (catenated dimers, CDs) could be assessed by gel electrophoresis: CDs structures could only derive from a cohesin conversion mechanism in yeast cells where *de novo* loading is not functional. Using this smart assay system Nasmyth and colleagues have demonstrated that both mechanisms exist in budding yeast cells, but they require different sets of protein factors. In fact, the cohesin conversion mechanism requires Tof1, Csm3, Chl1 and Ctf4 (the so-called TCCC pathway), but not Scc2, whereas the *de novo* loading process requires Mrc1, the RFC^Ctf18^ alternative PCNA clamp loader, in addition to Scc2 [42]. Nevertheless, the possibility that other essential replisome components are also directly involved in these cohesion establishment pathways cannot be excluded. However, addressing this issue would require the discovery of specific separation-of-function mutant alleles of these essential replication factors that are defective only in cohesion establishment but not in DNA replication.

## 5. Smc3 Modification by Acetyltransferases at the Replication Fork

**Figure 2:**
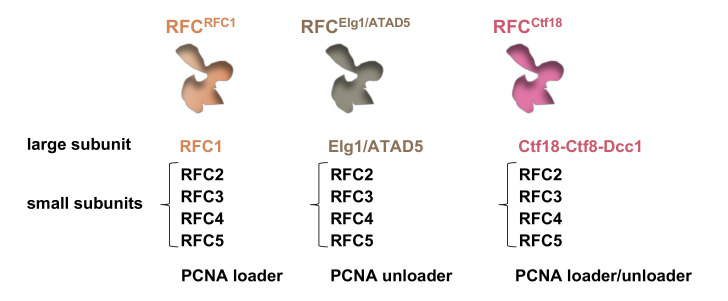
Replication factor C and its alternative forms involved in chromosomal cohesion. Subunit composition and role of each factor are reported. RFC^Ctf18^ contains Ctf8 and Dcc1 as two additional subunits.

As previously mentioned, a critical step during cohesion establishment at the replication forks is the acetylation of the Smc3 cohesin subunit. This modification renders cohesin unable to hydrolyze ATP and to undergo those conformational changes that enable ring opening and DNA release [52]. Thus, in order to ensure that only sister chromatids, but no other unrelated DNA molecules, are topologically entrapped by the cohesin rings, it is crucial that acetylation is executed in the context of the replication forks and that the dedicated acetyltransferases are stably associated with the ongoing replisomes. An important role in recruiting these enzymes to the DNA replication machinery is played by the PCNA factor. PCNA is the homo-trimeric ring-like sliding clamp that acts mainly as a processivity factor for the replicative DNA polymerases, but also coordinates a myriad of interactions between replication and other processes [53]. It was shown that in budding yeast the acetyltransferase Eco1 associates with the replication forks by directly binding PCNA. This interaction is mediated by a PCNA-interacting protein (PIP) box of Eco1 that appears to be conserved in Esco1 and Esco2, the Eco1 mammalian orthologs [53,54]. In line with these findings, overexpression of PCNA rescues temperature-sensitive mutants of *ECO1* in budding yeast [55]. During S phase, PCNA is loaded onto DNA by the action of the replication factor C (RFC). The latter, which has ATPase activity and belongs to the ATPases associated with various cellular activities (AAA^+^) family, is composed of one large (Rfc1) and four small subunits (Rfc2-Rfc5) [56]. Several Rfc1 paralogs were identified (Elg1/ATAD5, Rad24/Rad17 and Ctf18) that define alternative forms of the replication factor C with different functions [57]. While the “canonical” RFC complex (RFC^Rfc1^) is responsible for loading PCNA onto the lagging strand to provide a processivity clamp to DNA polymerase δ, RFC^Elg1/ATAD5^ has been demonstrated to promote release of PCNA from the lagging strand after Okazaki fragment maturation [58,59]. Another alternative version of RFC, which contains Ctf18, Ctf8 and Dcc1 (named RFC^Ctf18^), can load and unload PCNA onto DNA supporting cohesin acetylation and sister chromatid cohesion establishment in either yeast or mammalian cells [51,60,61,62] (see Figure 2).

In Ctf18-depleted yeast cells, the level of PCNA associated with chromatin is remarkably reduced but replication and cohesion establishment still take place, indicating that this alternative clamp loader is not essential for cohesin acetylation to occur at the replication forks. In a very recent work carried out by the Uhlmann group, the functions of these alternative versions of the replication factor C have been investigated in *S. cerevisiae* [63]. This biochemical study has revealed for the first time the existence of an even distribution of PCNA between the leading and lagging strand. This finding is unexpected considering that the PCNA sliding clamp function is mainly required on the lagging strand to promote elongation of the Okazaki fragments by DNA polymerase δ. Two mechanisms were discovered by Uhlmann and colleagues that balance PCNA levels between the two replication fork strands. On one hand, RFC^Ctf18^ promotes PCNA loading mainly onto the leading strand; on the other hand, release of PCNA from the lagging strand is executed by RFC^Elg1/ATAD5^ after Okazaki fragment ligation is completed. An additional important finding of this paper is that Eco1 acetylates cohesin in the wake of the replication forks and after Okazaki fragment maturation. Besides, the authors have also demonstrated the crucial role played by PCNA in recruiting Eco1 at the replication fork through a PIP box-mediated interaction [63]. Actually, the presence of a functional PIP box in the Eco1 polypeptide chain was controversial, due to the fact that this sequence is only partially conserved and attempts to co-immuno-precipitate PCNA and Eco1 from cell extracts were unsuccessful in many different laboratories. Uhlmann and collaborators have demonstrated that amino acid changes in the Eco1 putative PIP box result in remarkable sister chromatid cohesion defects in line with previous reports [54]. In addition, fusing an Eco1 version that contains a mutated PIP-box (named Eco1^-PIP^) to PCNA or to Fen1, the endonuclease involved in Okazaki fragment remodeling, rescued cell growth and sister chromatid cohesion defects of Eco1-depleted cells; in contrast, fusing Eco1^-PIP^ to other DNA replication factors (such as some subunits of the CMG complex or Ctf4 or Ctf18) did not exert the same effect. This suggests that in yeast cells Eco1 needs to be recruited behind the replication forks and on lagging strand to promote cohesion establishment [63].

**Figure 3:**
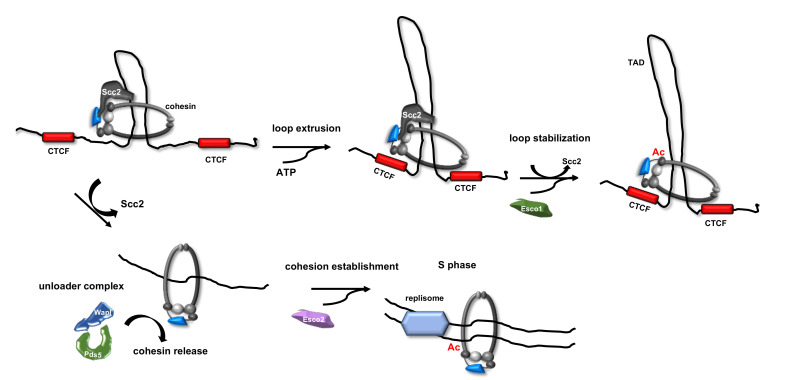
Regulation of cohesin functions by Esco1 or Esco2 acetyltransferases. Cohesin loading onto chromatin and loop extrusion activity both depend on association with Scc2 factor. Formation of chromatin topologically associating domains (TADs) in interphase cells is due to the loop extrusion activity of the cohesin-Scc2 complex. TAD boundaries are defined by the CCCTC-binding factor (CTCF), a sequence specific DNA binding protein that acts as a chromatin insulator [28]. The activity of Esco1 during interphase, before DNA replication starts, stabilizes TADs to define chromatin spatial organization. Conversely, Esco2 activity during S phase ensures that replicated sister chromatids are stably entrapped within the cohesin rings. Mode of DNA binding of cohesin-Scc2 complex is only indicative.

The different functions of Esco1 and Esco2 in promoting cohesin acetylation in human cells have not yet been completely elucidated. In the *Xenopus* egg extract system, Esco2 depletion results in cohesion loss, a phenotype that was not corrected by supplementing the extracts with recombinant Esco1 [64]. In cultured human somatic cells, the co-depletion of Esco1 and Esco2 caused cohesion anomalies that were more severe than the corresponding single depletions, demonstrating that the two proteins operate in parallel cohesion establishment pathways [65]. Of note, levels of expression of Esco1 and Esco2 differ during the different phases of the cell cycle. In fact, while Esco1 is present throughout the cell cycle at equal levels; Esco2 expression is low in G1 but peaks in S phase [64,65,66]. It was proposed that Smc3 acetylation is carried out by both Esco1 and Esco2 from S phase till mitosis in mammalian cells [67,68]. In contrast, Smc3 modification that occurs during G1 is only mediated by Esco1, because Esco2 is absent during this phase of the cell cycle [69,70,71,72]. Besides, the Rankin laboratory demonstrated that cohesion establishment requires mainly Esco2 in human cells, while the contribution of Esco1 was proposed to be very limited, even if most of the Smc3 acetylation was found to be Esco1-dependent [69]. Based on these findings Rankin and colleagues proposed that Esco1 and Esco2 regulate different cohesin functions: Esco2 is dedicated to sister chromatid pairing, while Esco1 modulates other nonconventional activities of the cohesin complex (such as maintenance of chromosome architecture and transcription regulation through chromatid loop formation and stabilization). This proposal is consistent with the finding that Esco1, but not Esco2, co-localizes with the insulator factor CTCF and cohesin at the base of chromatin loops and Esco1 loss leads to gene transcription dysregulation in human somatic cells (see Figure 3) [70,71].

**Figure 4:**
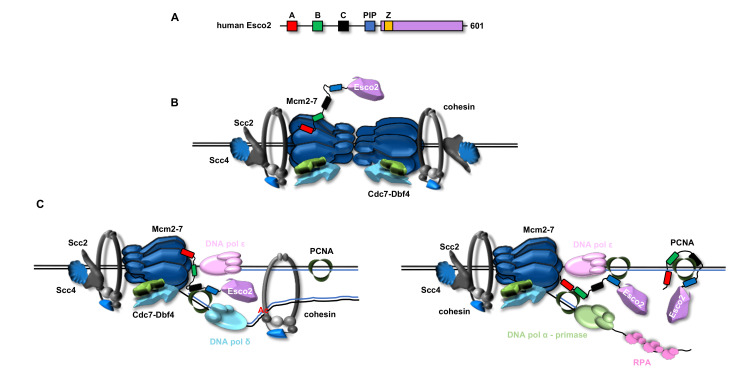
Recruitment of Esco2 at the replication forks in mammalian cells. (**A**) Schematic representation of the human Esco2 polypeptide chain. The conserved A, B, C and PIP boxes in the Esco2 N-terminal portion are indicated in different colours. *Z* stands for zinc finger motif (*orange box*). (**B**) Esco2 is recruited at the pre-RC through a direct interaction with the MCM2-7 complex mediated by box A (*red*). (**C**) During the elongation phase of DNA replication, Esco2 establishes multiple interactions with PCNA and/or the MCM complex either on the leading or the lagging strand through its conserved N-terminal sequences motifs (*red*, *green*, *black* and *blue rectangles*). Many DNA replication factors have been omitted for the sake of clarity. See text for details.

While, as previously described, in yeast cells the protein factor responsible for recruiting Eco1 to the replication fork is PCNA [53,63], a work carried out by Peters and collaborators revealed that Esco2 is associated with the DNA replication machinery through a direct interaction with the MCM complex in human cells [73]. This finding was confirmed by results of iPOND and Ch-IP seq experiments demonstrating that Esco2 is bound not only to the pre-replication complexes (pre-RCs), before DNA synthesis starts, but also to the ongoing replisomes, after replication origins are fired. Peters and collaborators highlighted the importance of an evolutionarily conserved sequence motif (named box A) in the N-terminus of Esco2 for mediating a direct interaction with MCM (in particular with the MCM4 and MCM7 subunits). In fact, an *Esco2* mutant allele, where box A was deleted by CRISPR-based gene editing, produced severe centromeric cohesion defects and displayed reduced association of Esco2 to chromatin and to the MCM complex in HeLa cells. Interestingly, in this cell line Smc3 acetylation was also compromised (following Esco1 depletion by RNA interfering), suggesting that recruitment of Esco2 to the replisomes via interaction with the MCM complex is crucial for cohesin acetylation at the replication fork [73]. Thus, according to this study, the recruitment of the acetyltransferase to the DNA replication machinery depends on MCM2-7 in human cells and is not mediated by PCNA, as observed in budding yeast [53,63]. This controversy seems to have been resolved in a recent interesting biochemical study showing that human Esco2 can establish multiple direct interactions with the DNA replication machinery through several highly conserved sequence motifs located in its unstructured N-terminal tail [74]. A multiple alignment of Esco2 sequences from various vertebrates has pinpointed four nearly invariant boxes shared by all the species analyzed. These include boxes A and B, previously found to be important for Esco2 chromatin binding (with box A corresponding to the motif reported to be critical for interaction with MCM by Peters and colleagues [73]), as well as a box C and an already known PIP box (see Figure 4A) [66,75]. Rankin and colleagues have shown that deleting each one of these conserved Esco2 boxes gives rise to reduced Smc3 acetylation and sister chromatid cohesion loss in human cells [74]. Besides, analyses based on GST-pulldown assays and plasmon resonance measurements have revealed that the above Esco2 conserved sequence motifs bind PCNA and other replication factors with different affinity. Thus, Rankin and collaborators have elaborated a model that reconciling these findings with the report by Peters and collaborators [73] predicts that Esco2 is initially recruited to chromatin by binding to MCM2-7 at pre-RCs; then, after origin licensing and replication fork establishment, Esco2 is engaged in a simultaneous multivalent interaction with PCNA (and/or with other replication factors including the MCM complex) through the above conserved sequence boxes located in the N-terminal part of its polypeptide chain (see Figure 4B,C).

## 6. Cohesin Conversion and *De Novo* Loading Mechanisms at the Replication Forks

**Figure 5:**
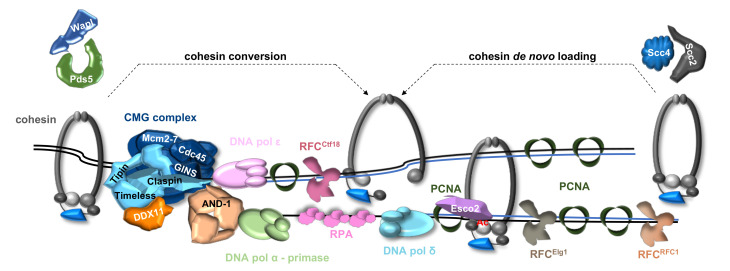
A hypothetical model of cohesion establishment at the human DNA replication fork. Some protein factors are omitted for simplicity (MCM10, Sororin, Fen1 and Dna2). Involvement of Pds5-Wapl and Scc2-Scc4 in cohesin conversion and *de novo* loading mechanism, respectively, has not yet been formally demonstrated in mammalian cells (see text for details). *Ac* stands for acetylation. Folding back of the lagging strand, proposed to enable DNA polymerase coupling, is not depicted here for simplicity. Relative position of the indicated protein factors is based on the structure of the yeast replisome proposed by Yeeles and colleagues [77].

The co-existence of cohesin conversion and *de novo* loading pathways appears to be a feature conserved in all eukaryotic cells, considering that genetic inactivation of a number of replication factors, shown to be involved in these pathways in budding yeast, also results in cohesion loss in vertebrate cells [37,76] (see Figure 5).

As pointed out in Section 4, the list of “cohesion establishment factors” includes the components of the so-called fork-protection complex (FPC) (Timeless/Tof1/Swi1, Tipin/Csm3/Swi3 and Claspin/Mrc1). The FPC has many interconnected functions that are important for preserving genome integrity during DNA replication. First of all, the FPC acts as a mediator of the S phase checkpoint promoting Chk1 phosphorylation by ATR during the DNA damage response. Second, the FPC, bridging the Cdc45/MCM2-7/GINS (CMG) complex with the replicative DNA polymerases, prevents uncoupling between DNA unwinding and synthesis at damaged sites or at difficult-to-replicate templates. Lastly, components of the FPC were demonstrated to promote chromosomal cohesion establishment in various systems, including *Caenorhabditis elegans* [78], *Xenopus laevis* egg extracts [79,80] and human cells [48,49]. In a recent work, it was shown that in human cells Timeless directly interacts with DDX11 (the human ortholog of yeast Chl1 protein) and this interaction is critical for recruiting cohesin to the replication forks and for establishing sister chromatid cohesion, since Timeless-defective binding *DDX11* mutant alleles were unable to correct the cohesion loss phenotype observed in DDX11-deficient HeLa cells [81]. The importance of Timeless, Tipin, DDX11 and AND-1/WDHD1 in sister chromatid cohesion establishment in human cells has been also reported in a recent work carried out by the Yu laboratory [33]. The precise roles played by these “cohesion establishment factors” have not yet been elucidated and it is not clear if they are only involved in assisting/promoting cohesin modification by the acetyltransferases or if they participate in any way in the cohesin re-modeling process that operates at the replication forks. Three possible fates of cohesin molecules that are pre-loaded on the un-replicated DNA template can be envisaged (see Figure 5). In the first scenario the replication machinery slides through pre-bound cohesin rings so that the newly copied DNA strands are passively entrapped within them. The second possibility is that the pre-loaded cohesin rings are transiently opened and transferred behind the replication forks to establish cohesion in the wake of the replication forks. The third option is that cohesin rings are dislodged by the advancing replisomes and released into the nucleoplasm. This latter mechanism is not consistent with studies carried out in yeast [42,82] and human cells [83] reporting a clear evidence that a cohesin “re-cycling” mechanism does take place at the replication forks. Besides, structural studies have revealed that the DNA replication machinery is too big to slide inside a cohesin molecule pre-loaded onto an un-replicated DNA template. In fact, the cohesin ring has a maximal diameter of about 40 nm (it is not larger than 20 nm in DNA-bound state), while the MCM2-7 complex, a single component of the replisome, has a diameter of about 11 nm. Thus, it appears more plausible that cohesin molecules bound to the un-replicated DNA are re-modelled and re-loaded behind the ongoing replisomes. It has been proposed that cohesin ubiquitylation, executed by the Rsp5 E3 ubiquitin ligase associated to the arrestin-like adaptor Bul2, is a critical modification required to trigger cohesin re-shaping by the Cdc48 ubiquitin-dependent unfoldase/segregase in budding yeast cells [82]. Cdc48 is involved in the displacement of many ubiquitylated proteins from chromatin, including the CMG complex, the Ku70-Ku80 heterodimeric DNA repair factor or RNA polymerase II in yeast [84]. Thus, it is possible that Cdc48 also participate in displacing cohesin from the un-replicated DNA template to assist ring opening mediated by Wapl. However, it remains to be established if this ubiquitylation-dependent mechanism of cohesin eviction is also present in metazoan cells and if it also operates in unperturbed normal S phase and not only during the DNA damage response. Of note, a Wapl-mediated displacement of cohesin has been recently reported to take place at stalled replication forks even in various human cell lines, where replication stress was induced either by hydroxyurea treatment or by oncogene activation [85].

## 7. Conclusions and Outlook

The molecular mechanisms underlying the functional connection between chromosomal cohesion and replication are starting to be unveiled. A complex picture is emerging where a plethora of protein factors come into play in an intricate network of physical and functional interactions. This represents a very fascinating research field that will certainly benefit from multidisciplinary complementary experimental approaches, including biophysical studies to visualize protein-DNA interactions in real time at the single-molecule level; structural analyses to define the action mechanisms of relevant protein and enzymes and in vivo experiments using genetically engineered cell and animal model systems to explore the physiological roles and examine the impact of loss/mutation of these protein factors. Studies based on all these complementary experimental approaches will help clarify the following major open questions in the field: (1) how the cohesin-Scc2 complex switches from a DNA loop extrusion to a DNA tethering mode? (2) Is the Scc2/NIPBL-Scc4 loader complex associated with the ongoing replisomes? (3) How is cohesin recycled or *de novo* loaded at the replication forks? (4) Which are the roles played by the cohesion establishment factors in these processes? (5) How is the activity of the different clamp loader complexes regulated on the leading and lagging strand? How are Esco1 and Esco2 functions regulated?

## Figures and Tables

**Table 1:** DNA replication factors involved in chromosomal cohesion establishment.

Replication Protein		Function
*S. cerevisiae*	*H. sapiens*	
Chl1 [43,44]	DDX11/ChlR1 [45,46]	DNA helicase
Tof1 [43,47]	Tim/Timeless [48,49]	S phase checkpoint/fork protection
Csm3 [43,47]	Tipin [48,49]	S phase checkpoint/fork protection
Mrc1 [43,47]	Claspin [49]	S phase checkpoint/fork protection
Ctf4 [50]	AND-1/WDHD1 [33]	DNA pol α - CMG link
RPA [13]	RPA [33]	Single-stranded DNA binding protein
Ctf18 [51]	Ctf18 ^1^	PCNA clamp loader subunit

^1^ The role of human Ctf18 in sister chromatid cohesion has not yet been investigated.
